# Psychometric assessment of a scale to measure bonding workplace social capital

**DOI:** 10.1371/journal.pone.0179461

**Published:** 2017-06-29

**Authors:** Hisashi Eguchi, Akizumi Tsutsumi, Akiomi Inoue, Yuko Odagiri

**Affiliations:** 1 Department of Public Health, Kitasato University School of Medicine, Kanagawa, Japan; 2 Department of Mental Health, Institute of Industrial Ecological Sciences, University of Occupational and Environmental Health, Japan, Kitakyushu, Japan; 3 Department of Preventive Medicine and Public Health, Tokyo Medical University, Tokyo, Japan; Hamamatsu Ika Daigaku, JAPAN

## Abstract

**Objectives:**

Workplace social capital (WSC) has attracted increasing attention as an organizational and psychosocial factor related to worker health. This study aimed to assess the psychometric properties of a newly developed WSC scale for use in work environments, where bonding social capital is important.

**Methods:**

We assessed the psychometric properties of a newly developed 6-item scale to measure bonding WSC using two data sources. Participants were 1,650 randomly selected workers who completed an online survey. Exploratory factor analyses were conducted. We examined the item–item and item–total correlations, internal consistency, and associations between scale scores and a previous 8-item measure of WSC. We evaluated test–retest reliability by repeating the survey with 900 of the respondents 2 weeks later. The overall scale reliability was quantified by an intraclass coefficient and the standard error of measurement. We evaluated convergent validity by examining the association with several relevant workplace psychosocial factors using a dataset from workers employed by an electrical components company (n = 2,975).

**Results:**

The scale was unidimensional. The item–item and item–total correlations ranged from 0.52 to 0.78 (p < 0.01) and from 0.79 to 0.89 (p < 0.01), respectively. Internal consistency was good (Cronbach’s α coefficient: 0.93). The correlation with the 8-item scale indicated high criterion validity (r = 0.81) and the scale showed high test–retest reliability (r = 0.74, p < 0.01). The intraclass coefficient and standard error of measurement were 0.74 (95% confidence intervals: 0.71–0.77) and 4.04 (95% confidence intervals: 1.86–6.20), respectively. Correlations with relevant workplace psychosocial factors showed convergent validity.

**Conclusions:**

The results confirmed that the newly developed WSC scale has adequate psychometric properties.

## Introduction

Workplace social capital (WSC) has attracted increasing attention as an organizational and psychosocial factor related to workers' health. There are several aspects of WSC, such as the extent and intensity of associational links or activities, and perceptions of support, reciprocity, and trust in the workplace [1]. A growing body of research has identified WSC as a determinant of employee health [2]. Empirical studies have found associations between low WSC and depression [3, 4], hypertension [5, 6], poor self-rated health [4, 7], smoking [4, 8], and high mortality [9]. On the other hand, a meta-analysis showed no association between most social capital dimensions and all-cause mortality, cardiovascular disease or cancer [10]. Thus, further studies are necessary to explore the health effects of WSC.

Several WSC scales have been used in Western countries. Trust and mutual aid in the workplace have been measured in the United States [11]. A 6-item scale was developed in Germany to examine personal aspects of social capital, such as shared values, support, aggregation, and recognition of trust within an organization [12, 13]. In Finland, Kouvonen et al. developed an 8-item scale to measure a multifaceted concept that included reciprocity, mutual aid, and cooperative interpersonal relationships beyond differences in workplace position [14]. Currently, Kouvonen et al.’s scale is the most frequently cited instrument in the occupational stress research field [6, 9, 15].

The separate and combined effects of the three types of social capital (i.e., bonding, bridging, and linking social capital) have attracted broad interest [16]. Bonding refers to the value assigned to social relationships among individuals with similar socioeconomic attributes. Bridging describes relationships between dissimilar persons at the same hierarchical levels. Linking refers to relationships between persons across different hierarchical levels. These constructs are relevant in specifying how WSC inheres in relationships between individuals in similar social contexts and different levels of society [14, 16].

Bonding WSC is of particular importance in Japanese society because Japanese culture is group-oriented; altruism, teamwork, and group cohesiveness are all strongly emphasized in Japanese society, and individual identity is defined by the social group [17–19]. Trust and reciprocity are a key factor in Japanese society. Previous studies from Japan showed the association of trust and reciprocity with worker’s health [4, 20]. The use of well-validated questionnaires is indispensable for measurement across diverse cultural settings. However, there are currently no original scales that measure bonding, trust and reciprocity of WSC in consideration of Japanese context.

This study aimed to analyze a newly developed scale for measuring bonding WSC and to assess the psychometric properties in a Japanese working population. The development of the scale was based on the COnsensus-based Standards for the selection of health Measurement INstruments (COSMIN) [21].

## Materials and methods

### Scale development

The measurement theory underlying the scale development was based on the reflective model (COSMIN Box E-1). Fourteen items were generated for the scale during the process of developing the New Brief Job Stress Questionnaire [22]. These items were collected from previous questionnaires based on relevant occupational stress theories and through a series of meetings with experts and stakeholders. The stakeholder meeting comprised researchers from five institutes or departments of occupational safety and health, occupational health personnel (physicians, nurses, and hygienists), and representatives of two employer associations and one employee organization. As previously mentioned, bonding WSC is of particular relevance to Japanese society [23]; therefore, we selected items relevant to bonding WSC to measure the network, trust, and reciprocity aspects of WSC. The items assessing the network aspects of WSC were adapted from Kouvonen et al. [14] and included three items that measured bonding WSC. The trust and reciprocity items were taken from previous Japanese studies that used the social cohesion approach to conceptualizing social capital [4, 11, 19, 20, 24–26]. These studies examined individuals’ perceptions of the trustworthiness of others, as well as the norms of solidarity and reciprocity that exist within the groups to which individuals belong. Norms and social trust represent features of social relationships that facilitate collective action for mutual benefit at workplaces [11]. Content validity, including face validity, was checked via item analyses and stakeholder discussions, and a consensus was reached that six items would be retained for the scale. All items were answered using a 4-point scale (1 = strongly disagree to 4 = strongly agree). Ratings for all items were summed to provide a single index. The scale is the following (COSMIN Box A-1, D-1, D-3, D-4).

Item 1. People keep each other informed about work-related issues in the work unit.
Item 2. We have a “we are together” attitude.
Item 3. People feel understood and accepted by each other.
Item 4. In our workplace, there is an atmosphere of helping each other.
Item 5. In our workplace, we trust each other.
Item 6. Our workplace is a place of laughter and smiles.

### Study design and population

#### Online survey

We conducted a new survey to confirm the reliability and validity of the WSC scale. A series of online surveys was conducted in February 2016 with participants registered with a Japanese online survey company. In total, 581,660 working people (excluding those who were self-employed, unemployed, or students) aged 20–69 years, of both sexes and all ages, were randomly invited to participate in the survey. Participants were selected with a random number generator; the study population comprised individuals interested in participating in a survey that provided a financial incentive for responding. The online survey company contacted selected registrants to answer the survey and ceased recruitment when the total number of participants reached the target number. The sex ratio was 1:1 and there were an equal number of participants in each age group (20–29, 30–39, 40–49, 50–59, and 60–69 years). The financial incentive for participants was modest, valued at approximately a few US dollars. For economic reasons, recruitment ceased when the number of participants exceeded 1,650; this was a moderate sample size based on a previous study [27] (COSMIN Box A-4, A-6, B-3, C-3, E-4, H-3, J-3). At least 301 participants were considered necessary to detect an intraclass correlation efficient (ICC) (2,1) ≥ 0.50 (error α = 0.05 and β = 0.20) between the first and second survey[28]. We repeated the survey with 900 of the respondents using the same WSC questionnaire 2 weeks later (COSMIN Box B-4, B-5, B-6, B-9, C-4, C-5, C-6, C-9). The second survey respondents were recruited on a first-come, first-served basis until the number of participants achieved 900. A time interval of about 2 weeks is considered appropriate for the evaluation of instruments [29] (COSMIN Box B-8, C-8). There were no differences in WSC and participant demographic characteristics between the two survey periods (COSMIN Box Generalisability-4, -5, -7). Because our online survey required participants to answer all the questions, no participants had missing items (COSMIN Box A-2, A-3, B-1, B-2, C-1, C-2, H-1, H-2, Generalisability-8).

#### Company survey

We used the existing database to confirm the convergent validity of the WSC scale. We used part of a cross-sectional dataset collected from a baseline survey of an occupational cohort study on social class and health in Japan (Japanese Study of Health, Occupation, and Psychosocial Factors Related Equity: J-HOPE), which was conducted from April to June 2011. We selected workers employed by an electrical components company at two manufacturing sites. The total number of employees in the company was about 15,000. This dataset comprised information from 3,461 workers who completed a self-administered questionnaire. This contained scales that measured a broad range of relevant workplace psychosocial factors and psychological distress together with the WSC scale (response rate = 95.3%). After excluding 486 workers who had at least one missing response on the questionnaire, the final number of respondents was 2,975 (2,204 men and 771 women: valid response rate = 86.0%), which was a moderate sample size based on a previous study [27] (COSMIN Box A-4, A-6, B-3, C-3, E-4, F-3, H-3, J-3). We compared the final respondents of the study population (n = 2,975) with participants with at least one missing response on the questionnaire (n = 486). There were no significant differences between these groups in job demands, coworker support, effort–reward imbalance (ERI), penetration of management philosophy, work engagement, psychological distress, or education. However, job control, supervisor support, procedural justice, interactional justice, the proportion of men, number of younger individuals, number of regular workers, and number of less skilled workers were lower in the missing response group (COSMIN Box Generalisability-4, -5, -7, -8). Among participants with at least one missing response on the WSC scale, 69% had only one missing response. There were no large differences in the number of missing responses between individual items related to WSC: item 1 (n = 14), item 2 (n = 19), item 3 (n = 18), item 4 (n = 16), item 5 (n = 17), and item 6 (n = 13) (COSMIN Box F-2).

### Measures

#### Online survey

**Kouvonen et al.’s [14] WSC scale:**To evaluate criterion validity, we used a Japanese version of Kouvonen et al.’s WSC scale [14] (COSMIN Box H-4). This measure is a reliable and valid indicator of WSC, and shows strong associations with self-rated health [14], depression [30], hypertension [6], and mortality [9]. Using a 5-point Likert scale, participants report their perceptions of the following issues: “We have a ‘we are together’ attitude,” “People feel understood and accepted by each other,” “People keep each other informed about work-related issues in the work unit,” “Members of the work unit build on each other’s ideas in order to achieve the best possible outcome,” “People in the work unit cooperate in order to help develop and apply new ideas,” “We can trust our supervisor,” “Our supervisor treats us with kindness and consideration,” and “Our supervisor shows concern for our rights as an employee.” Odagiri et al. [31] have found acceptable internal consistency, reliability, and validity for the Japanese version of the WSC. In our online survey, Cronbach's α coefficient was 0.94.

#### Company survey

**Job demands, job control, supervisor support, and coworker support:** We used the scales measuring job demands, job control, supervisor support, and coworker support from the Japanese version of the Job Content Questionnaire [32]. The Job Content Questionnaire, developed by Karasek, is based on the job demands–control (or demand–control–support) model and includes scales for job demands (five items), job control (nine items), supervisor support (four items), and coworker support (four items) rated on a 4-point scale (1 = strongly disagree to 4 = strongly agree). The reliability and validity of the Japanese version of the Job Content Questionnaire are acceptable, as shown by Kawakami et al. [33]. In the present study sample, Cronbach’s α coefficients were 0.68, 0.82, 0.90, and 0.79 for the job demands, job control, supervisor support, and coworker support scales, respectively (COSMIN Box F-7, F-8).

#### Effort–reward imbalance (ERI)

To assess ERI, we used data collected from a simplified Japanese version of the ERI Questionnaire [34]. This version includes scales for effort (three items) and reward (seven items) rated on a 4-point scale (1 = strongly disagree to 4 = strongly agree). Kurioka et al. [35] have reported acceptable reliability and validity for the simplified Japanese version of the ERI Questionnaire. In the present study sample, Cronbach’s α coefficients were 0.78 and 0.72 for the effort and reward scales, respectively. We calculated the effort/reward ratio to measure the extent of the ERI (COSMIN Box F-7, F-8).

#### Procedural and interactional justice

Two aspects of organizational justice (i.e., procedural and interactional) were assessed using the Japanese version of the Organizational Justice Questionnaire. This instrument was developed by Moorman [36] and was modified by Elovanio et al. [37]. It comprises a 7-item scale that measures procedural justice, and a 6-item scale that measures interactional justice, both of which are rated on a 5-point scale (1 = strongly disagree to 5 = strongly agree). Inoue et al. [38] have confirmed the reliability and validity of the Japanese version of the scale. In the present study sample, Cronbach’s α coefficients were 0.88 and 0.94 for procedural and interactional justice scales, respectively (COSMIN Box F-7, F-8).

#### Penetration of management philosophy

Penetration of management philosophy was assessed using three questions derived from previous studies [39]. Participants reported their perceptions of the following issues: “I understand my company management philosophy," “My company management philosophy has a strong effect on my attitudes towards my work," and “My company management philosophy fits my sense of value." Responses were measured on a 5-point scale (1 = completely disagree to 5 = completely agree). Item scores were summed and greater scores indicated greater penetration of management philosophy. In the present study sample, Cronbach’s α coefficient was 0.86 for the penetration of management philosophy scale (COSMIN Box F-7, F-8).

#### Work engagement

Work engagement was assessed using the short form of the Utrecht Work Engagement Scale (UWES) [40]. The UWES includes three subscales that reflect the underlying dimensions of engagement: vigor, dedication, and absorption (three items for each). All items were scored on a 7-point Likert scale ranging from 0 (never) to 6 (always). The validation study of the Japanese version of the UWES [41] recommends that work engagement should be treated as a unitary construct owing to high correlations among the three components. In the present study sample, Cronbach’s α coefficient was 0.93 for the UWES (COSMIN Box F-7, F-8).

#### Psychological distress

The K6 scale, developed by Kessler et al. [42], consists of six items that measure the extent of psychological distress using a 5-point response option (0 = none of the time to 4 = all of the time). The Japanese version of the K6 scale has acceptable reliability and validity [43]. The item scores are summed to calculate a total score; higher scores indicate greater psychological distress. In the present study sample, Cronbach’s α coefficient was 0.88 for the K6 scale (COSMIN Box F-7, F-8).

#### Demographic variables

In both the online and company surveys, we measured sex, age group (18–29, 30–39, 40–49, 50–59, and 60–69 years), educational attainment (more than 12 years or not greater than 12 years), employment contract (regular or part-time), and job types to describe the study population (COSMIN Box D-2).

### Hypothesis testing

According to COSMIN, hypothesis testing must be used when a gold standard is not available. WSC is theoretically related to other psychosocial factors in the workplace [14]. We hypothesized that WSC might be negatively correlated with job demands (r ≤ −0.05), ERI (r ≤ −0.20), and psychological distress (r ≤ −0.05), and positively correlated with job control (r ≥ 0.20), procedural justice (r ≥ 0.50), interactional justice (r ≥ 0.50), work engagement (r ≥ 0.30), and penetration of management philosophy (r ≥ 0.30). These hypothesized effect sizes were estimated from previous studies [14, 44, 45] (COSMIN boxes F-4, F-5, F-6, F-7).

### Statistical analysis (COSMIN Box F-10)

#### Online survey

We used classical test theory to measure bonding WSC. Mean values and standard deviations were calculated for each item of the WSC scale. We tested sex, age, education, employment contract, and occupational differences in the WSC scale with analysis of variance. Item–item and item–total Spearman’s correlations were examined. Exploratory factor analyses were conducted, and all six items were entered using the unweighted least squares method. Factors with eigenvalues of more than 1.0 were extracted, and the Promax rotation method was used to obtain clear factorial structures (COSMIN Box E-6). The survey was repeated using the same WSC scale for 900 of the respondents after 2 weeks to assess the replicability of the WSC scale using the test–retest method (COSMIN Box B-4, B-5, B-6, B-9). The overall scale reliability was quantified by both an intraclass correlation coefficient (ICC) (2,1) based on a single measurement two-way random effects model of absolute agreement [46] (COSMIN Box B-11) and the standard error of measurement (SEM) (COSMIN Box C-11). ICC (2,1) was interpreted in accordance with published recommendations: an ICC (2,1) of 0.90 or higher was considered excellent, 0.75 or higher good, 0.50 or higher moderate, and lower than 0.50 poor [46]. The larger the SEM, the lower the test reliability and the less precision in the measures and scores obtained [47]. To evaluate internal consistency, Cronbach’s α coefficient was calculated (COSMIN Box A-7, A-9). To confirm criterion validity, Pearson’s correlation was computed between our scale scores and scores from Kouvonen et al.'s scale (COSMIN Box H-6).

#### Company survey

To evaluate convergent validity, we calculated the Pearson’s correlation coefficients between the WSC and job demands, job control, supervisor support, coworker support, ERI, procedural justice, interactional justice, penetration of management philosophy, work engagement, and psychological distress based on the hypothesis testing.

The level of significance used was 0.05 (two-tailed). IBM SPSS Statistics for Windows, Version 23 (IBM Corp., Armonk, NY, USA) was used for the statistical analyses.

### Ethics review and approval

The study aims and protocol were reviewed and approved by the Research Ethics Committee of the Graduate School of Medicine and Faculty of Medicine, The University of Tokyo (No. 2772), the Kitasato University Medical Ethics Organization (Nos. B12-103 and B15-132), and the Ethics Committee of the University of Occupational and Environmental Health, Japan (No. 10–004). Written consent was obtained from participants.

## Results

Participants’ demographic characteristics and means and standard deviations of total WSC score by the characteristics for the two databases are shown in Table 1 (COSMIN Box J-4, J-7, Generalisability-2). Means of total WSC were significantly different between ages, educations, and occupations in the online survey and between ages and occupations in the company survey.

**Table 1:** Participant demographics and means and standard deviations of total workplace social capital score.

	Online survey (n = 1,650)				A company survey (n = 2,975)			
	N	Mean	SD	p^a^	N	Mean	SD	p^a^
Sex								
Men	825	16.33	(4.01)	0.42	2204	16.91	(3.23)	0.58
Women	825	16.44	(4.15)		771	16.80	(3.58)	
Age								
18–29	330	16.42	(4.03)	0.01	763	16.95	(3.53)	0.01
30–39	330	16.06	(4.25)		792	16.58	(3.40)	
40–49	330	16.08	(4.03)		856	16.95	(3.18)	
50–59	330	16.28	(3.91)		480	17.04	(3.12)	
60-	330	17.06	(4.11)		84	17.65	(3.14)	
Education								
≤12 years	421	16.06	(4.00)	<0.01	1669	16.73	(3.40)	0.06
>12 years	1229	16.49	(4.10)		1306	17.08	(3.22)	
Employment contract								
Regular	985	16.25	(4.17)	0.81	2500	16.88	(3.28)	0.11
Part-time	665	16.58	(3.93)		475	16.92	(3.58)	
Occupation								
Managers	164	17.66	(3.94)	<0.01	271	17.93	(2.84)	<0.01
Professionals	198	16.85	(3.72)		205	16.91	(3.35)	
Technicians and associate professionals	101	16.32	(3.70)		352	16.97	(3.03)	
Clerical support workers	520	15.98	(4.12)		278	16.88	(3.44)	
Service and sales workers	317	16.55	(4.26)		24	16.00	(3.97)	
Craft and related trade workers	58	16.33	(4.12)		192	17.05	(3.35)	
Plant and machine operators, and assemblers	58	15.93	(3.60)		712	16.52	(3.24)	
Armed forces occupations	151	15.74	(4.09)		477	16.63	(3.72)	
Others	83	16.22	(4.35)		464	17.00	(3.26)	

SD, standard deviation.

^a^, Analysis of variance.

### Online survey

Table 2 shows the means, standard deviations, item–item and item–total correlations, and Cronbach's α coefficients of the scale if each item was deleted. The mean for each item was between 2.0 and 3.0. Correlations between items varied between 0.52 and 0.78 (p < 0.01). The item–total correlations ranged from 0.79 to 0.89 (p < 0.01). The WSC scale was unidimensional (principal component analysis; eigenvalue = 4.43) (COSMIN Box A-5). The correlations between the WSC scores of the two survey rounds indicated high test–retest reliability (r = 0.74, p < 0.01). The ICC (2,1) and SEM for WSC were 0.74 (95% confidence intervals [CI]: 0.71–0.77) and 4.04 (95% CI: 1.86–6.20). The mean of the time variation for WSC was 0.06 ± 2.92. The internal consistency of the scale was good, with a Cronbach's α coefficient of 0.93. The presence or removal of any of the items did not materially alter the internal reliability of the scale. The scale score was highly correlated with that of Kouvonen et al.’s scale (r = 0.81, p < 0.01).

**Table 2:** Means, standard deviations, item–item correlations, item–total correlations, and Cronbach’s α if item deleted: Social capital items measured in randomly selected workers by an online survey (N = 1,650).

	Means	SD	Item 1^b^	Item 2^b^	Item 3^b^	Item 4^b^	Item 5^b^	Item–total correlation^b^	Cronbach’s α if item deleted
Item 1.	2.81	(0.78)						0.86	0.91
Item 2.	2.68	(0.77)	0.72					0.89	0.91
Item 3.	2.73	(0.78)	0.65	0.67				0.83	0.92
Item 4.	2.75	(0.81)	0.71	0.71	0.67			0.89	0.91
Item 5.	2.61	(0.80)	0.67	0.78	0.66	0.73		0.88	0.91
Item 6.	2.81	(0.81)	0.59	0.60	0.52	0.64	0.63	0.79	0.93

SD, standard deviation.

^b^, Item–item correlations and item–total correlations: all Spearman correlations; p < 0.001 in all cases.

### Company survey

An aspect of convergent validity was tested by exploring the associations between the WSC score and the theoretically related constructs of job demands, job control, supervisor support, coworker support, ERI, procedural justice, interactional justice, penetration of management philosophy, work engagement, and psychological distress. Table 3 shows that, as expected, high WSC scores were significantly negatively associated with job demands, ERI, and psychological distress; and positively associated with job control, supervisor support, coworker support, procedural justice, interactional justice, penetration of management philosophy, and work engagement (all p < 0.01).

**Table 3:** Association between workplace social capital score and other constructs in workers employed at two manufacturing sites of an electrical components company (N = 2,975).

Other constructs	Correlation coefficients^a^
Job demands	−0.10
Job control	0.21
Supervisor support	0.45
Coworker support	0.63
Effort–reward imbalance	−0.24
Procedural justice	0.52
Interactional justice	0.51
Penetration of management philosophy	0.31
Work engagement	0.36
Psychological distress	−0.30

^a^Pearson’s correlations; p < 0.001 in all cases.

## Discussion

We assessed the psychometric properties of our new WSC scale, which was developed to reflect the importance of bonding social capital in work environments in Japan. Following the COSMIN model, we demonstrated acceptable levels of internal consistency (Box A), reliability (Box B), measurement error (Box C), content validity (Box D), structural validity (Box E), hypothesis testing (convergent validity) (Box F), criterion validity (Box H), and generalizability among diverse populations. However, we did not examine cross-cultural validity (Box G), responsiveness (Box I), or interpretability (Box J).

We examined bonding rather than bridging or linking social capital. As mentioned above, bonding is important in the cultural context of the Japanese workplace [23]. Bridging social capital is defined as respect and mutuality between people who know that they are not alike in some sociodemographic (or social identity) sense (differing by age, ethnic group, or socioeconomic class). Linking social capital refers to relationships among people who are interacting across explicit, formal, or institutionalized power or authority gradients, which might lead to top-down management in the workplace. In the previous study in the Japanese workplaces, the results of bonding WSC were consistent with previous studies, whereas linking and bridging WSC were not consistent with previous studies [48]. Because workers in Japan are from less diverse backgrounds (e.g., ethnic group or social class) and top-down management styles are less common than in Western countries [49], the bonding social capital might make more effect on the workers’ health than linking and bridging in Japanese workplaces.

Previous studies using our scale showed significant associations between WSC score and psychological distress, as expected [50, 51]. These results were consistent with previous studies using other WSC measures [3, 7] and provide evidence for the validity of our scale. However, the health benefits of social capital can be observed both at the individual and aggregated (workplace) level [14]. As our study only demonstrated psychometric properties at the individual level, further studies are needed to evaluate the association between workplace-level WSC and workers’ health.

The present study has some limitations. First, participants in the company survey were workers employed by an electrical components company at two manufacturing sites or internet users in Japan. It is unclear how much the reported psychometric properties can be generalized to other industries and non-internet users. Therefore, more research with participants of differing backgrounds is needed to validate the measure further. Second, our scale was constructed using a relatively small number of items. However, as the items were selected based on the conventional core concepts of social capital, such as "trust," "reciprocity,” and “collective efficacy” [4, 11, 19, 20, 24–26], as well as through discussion between experts, occupational health professionals and practitioners, we consider our new WSC scale to have a certain level of content validity. Third, this scale was originally developed in Japanese. We assumed that this scale was used in the Japanese workplace. However, there are some countries with similar working cultural background such as collectivism, allocentrism and non-diverse in east Asia[23, 52]. Therefore, this concise scale could be useful in work environments other than Japan to see the health effect of bonding workplace social capital by confirming the cross-cultural validity (COSMIN Box G). Fourth, COSMIN requires a further test for responsiveness, which refers to the ability of an instrument to detect change over time in the construct to be measured (COSMIN Box I). Fifth, we could not calculate the minimal important change or the minimal important difference, which is used to define meaningful change (COSMIN Box J-8).

## Conclusion

Although further validation tests are required, the findings show that the newly developed WSC scale has adequate psychometric properties. We expect this concise scale to be useful in measuring WSC at workplaces where bonding social capital is relevant.
